# Correlation of Heterogeneous Local Martensite Tetragonality and Carbon Distribution in High Carbon Steel

**DOI:** 10.3390/ma15196653

**Published:** 2022-09-25

**Authors:** Thomas Kohne, Alexander Dahlström, Aimo Winkelmann, Peter Hedström, Annika Borgenstam

**Affiliations:** 1 Department of Materials Science and Engineering, KTH Royal Institute of Technology, 10044 Stockholm, Sweden; 2 Academic Centre for Materials and Nanotechnology (ACMiN), AGH University of Science and Technology, 30059 Kraków, Poland

**Keywords:** martensite transformation, martensite tetragonality, atom probe tomography, electron backscatter diffraction

## Abstract

A novel approach for the correlation of local martensite tetragonality determined by electron backscatter diffraction and carbon distribution by atom probe tomography (APT) is presented. The two methods are correlated by site-specific sample preparation for APT based on the local tetragonality. This approach is used to investigate the local carbon distribution in high carbon steel with varying local martensite tetragonality. Regions with low tetragonality show clear agglomeration of carbon based on statistical nearest neighbour (NN) analysis, while regions with high tetragonality show only small elongated agglomerations of carbon and no significant clustering using NN analysis. The APT average bulk carbon content shows no quantitative difference between regions with low and high tetragonality, indicating that no significant long-range diffusion of carbon has taken place.

## 1. Introduction

The tetragonality of martensite in carbon steels and its relation to carbon content has been investigated for almost 100 years to improve the understanding of martensitic transformation and the effect of subsequent heat treatments, such as tempering [1,2,3,4,5,6,7,8,9]. The general linear relationship between the average bulk carbon content and average martensite tetragonality has been confirmed above 0.2 weight per cent (wt%) C, while the exact slope of the linear relationship [1,5,7,10,11], as well as the presence of tetragonality below 0.2 wt% C, are still under debate [7,9,12].

The tetragonality is typically determined by X-ray diffraction (XRD), but it is important to note that conventional XRD only measures an average tetragonality. Additionally, reliable tetragonality measurements by XRD can be challenging considering peak overlap, peak broadening interpretation and accurate fitting [8,13,14,15]. Furthermore, there are clear indications from XRD measurements that the tetragonality is heterogeneously distributed due to the presence of an additional third peak or asymmetric peak shapes [6,8,14]. The heterogeneous distribution of tetragonality is more evident for steels with a carbon content above 0.44 wt% due to an increased deviation from the expected body-centred tetragonal crystal structure with a single tetragonality in XRD measurements [8]. The results of previous in situ high-energy XRD experiments during the heat treatment for high carbon steels [14,15] have shown that the martensite tetragonality changes during transformation, leading to a heterogeneous distribution of tetragonality after quenching. The heterogeneous distribution of tetragonality has been confirmed by local tetragonality measurements using electron backscatter diffraction (EBSD) based on pattern matching approaches [8,13,16]. However, the relationship between the local tetragonality and local carbon content has not been studied previously.

Atom probe tomography (APT) allows the determination of the chemical content with near-atomic resolution, but the analysis of a microstructure with locally dispersed heterogeneous distributions within one phase can be challenging due to relatively small analysis volumes [17]. Previous APT work on high-carbon martensitic steels shows the agglomeration of carbon even for high cooling rates, but the relationship between local tetragonality and carbon content was not investigated [9]. Therefore, a new approach is applied to decide the position of the APT lift-out on the results of local tetragonality measurements by EBSD. This allows the correlation of the local tetragonality and carbon content on small length scales in high carbon steel.

## 2. Materials and Methods

**Figure A1:**
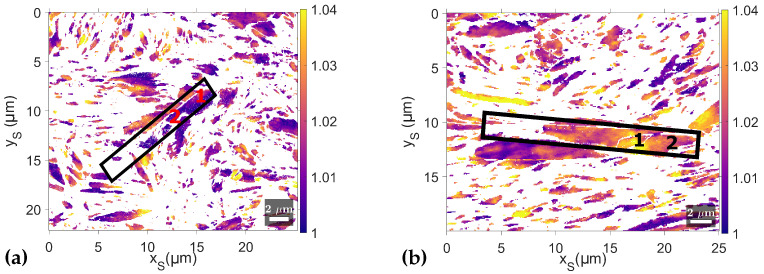
Local tetragonality map c/a determined by projective Kikuchi pattern matching [13,16] in EBSD with normalised cross-correlation coefficient *r* above 0.4. The white regions contain data points with a *r* value below 0.4 or are retained austenite. Lift-out regions (**a**) B and (**b**) C are marked by a black rectangle and the numbers 1 and 2 indicate the approximate positions of the APT tips, e.g., B1 or C2.

The composition of the studied steel was 1.2Mn-1.1Cr-0.2Si-0.74C-Fe(Bal.) in wt%, which corresponds to 3.3 atomic per cent (at%) C. The samples were cast, hot rolled at 1173 K, homogenized at 1473 K for 24 h and machined into rectangular dilatometer specimens. The heat treatment consisted of austenisation at 1233 K for 30 s with subsequent cooling with 30 K/s to 823 K and 15 K/s to 523 K, which is above the martensite start temperature. For the final cooling to room temperature (RT), two different cooling rates, 15 and 0.5 K/s were used. It has previously been observed that the microstructure consists of a mixture of martensite and retained austenite, with 19% retained austenite after cooling at a rate of 15 K/s and 21.7% after cooling at a rate of 0.5 K/s and a heterogeneous martensite tetragonality distribution was observed in all samples [14]. The sample with the final cooling rate of 15 K/s was chosen for further investigations due to the presence of large martensite units with both high and low tetragonality, see Figure 2b and Figure A1. Additionally, the 0.5 K/s sample was used as a reference for a low tetragonality region, see Figure 2a.

The investigation of the carbon distribution in an as-quenched martensitic steel is challenging due to the significant changes in carbon and tetragonality distribution during RT ageing [6,18]. In the present work, the studied samples were aged at RT for approximately 1.5–2 years; see Table 1 for exact ageing times. This RT ageing should generate a more stable state [6] and minimize the effect of time between local tetragonality and APT measurement. The influence of the different RT ageing times was estimated via the mean diffusion distance *x* determined by the Einstein equation $x=\sqrt{2Dt}$ with the diffusion coefficient *D* and time *t*. Due to the uncertainty in *D* for martensite [19,20,21,22,23], two different values were used, a lower $D_{l}=2.5\times 10^{-23}\;m^{2}/s$ [19] and an upper $D_{u}=2.0\times 10^{-21}\;m^{2}/s$ [20,21], to estimate *x*, and the resulting *x* values are shown in Table 1. For $D_{u}$, the $x_{u}$ values are between 446 and 535 nm, and for $D_{l}$, $x_{l}$ is between 50 and 60 nm. The difference for $x_{l}$ is quite small, 10 nm, while the absolute values for $x_{u}$ are significantly larger than the APT tip dimensions and clear differences in the average carbon content between APT tips due to long-range carbon diffusion should be observed, which was not the case as seen in Table 1.

The 200 martensite peaks of the 15 K/s sample were measured by XRD in the 57–72${}^{\circ }$ 2θ range with a 0.02${}^{\circ }$ step size and 17 s dwell time to verify the presence of tetragonality after RT ageing. The XRD measurements were carried out in a Siemens D5000 X-ray diffractometer with a graphite monochromator and a copper target with $K_{\alpha ,1}$ radiation and a wavelength of 1.5406 Å.

The APT tips were prepared by site-specific lift-out by focused ion beam (FIB) [24,25] based on the EBSD results of local martensite tetragonality after RT ageing. Prior to EBSD, the samples were mechanically polished with 9, 3 and 1 μm diamond polishing suspension followed by electropolishing at 12 V for 60 s in Struers A3 electrolyte. The electropolishing resulted in a slight etching of the surface.

The EBSD measurements and the lift-out preparation were carried out in an FEI Nova 600 NanoLab dual-beam system with an Oxford Instruments Symmetry CMOS detector. The raw EBSD patterns were recorded at 20 kV and 9.5 nA with an acquisition time of 10 ms and a resolution of 156 × 128 pixels. The local tetragonality of martensite was calculated with the projective Kikuchi pattern-matching algorithm from Winkelmann et al. [13,16] for patterns with a normalized cross-correlation coefficient *r* above 0.4 to exclude poor fitting. The EBSD investigation was split into two steps, starting with larger EBSD scans to identify interesting regions for lift-out, followed by higher magnification EBSD scans of identified regions with a step size of 75 nm for more detailed information. The local martensite tetragonality results were analysed in the MTEX toolbox [26] to determine the optimal position of the lift-outs depending on the tetragonality value. In total, lift-outs were taken from four regions, one from the reference 0.5 K/s (A) and three from 15 K/s (B-D), and two APT tips per lift-out region were measured. The regions were chosen based on their varying local tetragonality, with two having a relatively lower tetragonality, A and B, and two having a relatively higher tetragonality, C and D, see Table 1. The names of the APT tips are a combination of the lift-out region, A–D, and the lift-out position, 1 or 2 in Figure 2 and Figure A1, resulting in, e.g., B1 for the APT tip from position 1 in region B.

The APT tips were measured in a LEAP 3000X HR in voltage pulse mode for the 15 K/s samples and laser pulse mode for the 0.5 K/s reference samples for practical reasons. For voltage pulse mode, the tip temperature was 70 K, and 20% pulse fraction, 200 kHz pulse frequency and 0.2% target evaporation were used. For the laser pulse mode, the tip temperature was 50 K, and 0.25 nJ pulse energy, 200 kHz pulse frequency and 0.5% target evaporation was used. The results were reconstructed and analysed in the IVAS 3.8.0 software using the tip profile with a focus on the distribution of carbon. The correct analysis of carbon in APT can be challenging due to multiple factors such as, e.g., surface migration of carbon [27] as well as the presence of multiple events and dissociation of molecular ions and the resulting complexity in indexing of the mass spectrum peaks [28,29,30,31,32]. Therefore, consistent indexing of mass spectrum peaks was used for all samples to allow the comparison between measurements. The peaks at 6, 6.5, 12 and 13 Da were assigned to *C*, at 24 Da to $C_{2}$, at 18, 18.5, 36 and 37 Da to $C_{3}$, and at 24.5 Da to $C_{4}$. The 24 Da peak is expected to be a superposition of $C_{2}^{+}$ and $C_{4}^{2+}$ due to the presence of the 24.5 Da peak, but only $C_{2}^{+}$ was selected following the conventions [29,33,34]. In the laser pulse mode, additional peaks for $C_{4}^{+}$ at 48, 49 and 50 Da were detected. The fraction of molecular ions were significantly higher for the 0.5 K/s reference tips analysed in laser pulse mode with values between 0.60 and 0.62 compared to 0.33 and 0.37 for 15 K/s.

The APT tips were split into multiple regions of interest (ROI) with a fixed size of $40\times 40\times 60\;{\mathrm{nm}}^{3}$, and the number of ROI depended on the size of the analysed tip, ranging from 1 to 4, see Table 1. The iso-concentration surfaces for a carbon content of 1.5 at% and 6 at% C were calculated to visualize the distribution of volumes with depleted carbon content lower than 1.5 at% C, as well as the enriched carbon content higher than 6 at% C. The statistical analysis of the carbon was focused on the nearest neighbour (NN) distribution [35,36] in comparison with a binomial distribution for the given average bulk content in IVAS. The variation of the normalized NN distribution can be expressed as [37]:

(1) $$V=\sum_{n}\sqrt{{\left(I_{O,n}-I_{B,n}\right)}^{2}}$$

with $I_{O,n}$ as the observed NN distribution at distance *n* and $I_{B,n}$ as the binomial distribution for the ROI’s average bulk content. The variation is a measurement of the statistical distance between ions, which can have a value between 0 and 2, with 0 being a perfect overlap of $I_{O,n}$ and $I_{B,n}$ while 2 is no overlap. The NN analysis was performed together for all carbon ions, including the molecular ions, to determine the general statistical distribution. The molecular ions were counted as a single neighbour due to the missing spatial information [17,34].

## 3. Results

### 3.1. XRD

**Figure 1:**
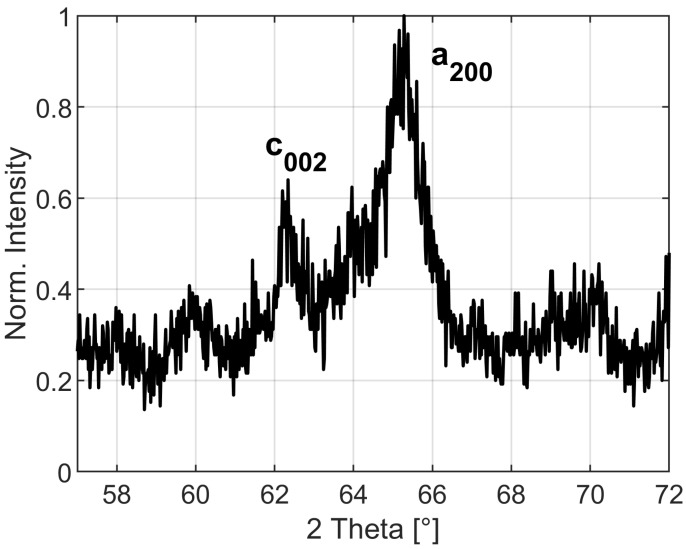
XRD measurement of $a_{200}$ and $c_{002}$ martensite peaks with possible carbide peaks at 60${}^{\circ }$ and 70${}^{\circ }$.

The XRD measurement of the 15 K/s sample has two clearly separated martensite peaks, $a_{200}$ and $c_{002}$, showing the presence of high tetragonality martensite after RT ageing, see Figure 1. In addition, $a_{200}$ has an asymmetric peak shape indicating a heterogeneous distribution of martensite tetragonality. Furthermore, possible carbide peaks with low intensities can be observed at 60${}^{\circ }$ and 70${}^{\circ }$.

### 3.2. APT Preparation

**Figure 2:**
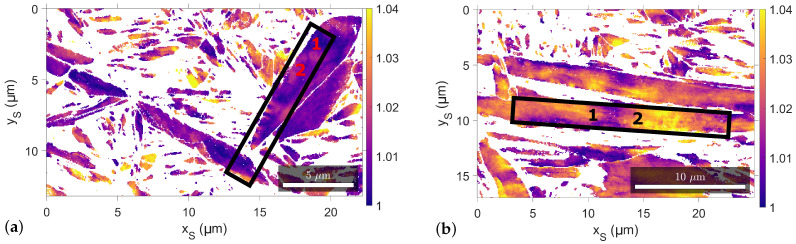
Local tetragonality c/a map determined by projective Kikuchi pattern matching [13,16] in EBSDwith a normalized cross-correlation coefficient *r* above 0.4. The white regions contain data points with an *r* value below 0.4 or are retained austenite. Lift-out regions (**a**) A and (**b**) D are marked by black rectangles, and the numbers 1 and 2 indicate the approximate positions of the APT tips, e.g., A1 or D2.

The local martensite tetragonality maps for both samples, 15 K/s and 0.5 K/s, determined by projective Kikuchi pattern matching [13,16] are shown in Figure 2, and a heterogeneous distribution is observed in both. The black rectangles mark the approximate position of lift-out regions A and D with the numbers indicating the expected position of the respective tip, e.g., A1 or D2, after preparation by FIB milling. The approximate lift-out regions are determined by the correlation of SEM images of the slightly etched surface and the EBSD scans due to the typical spatial distortion of EBSD maps [38,39,40,41]. The local tetragonality maps for lift-out regions B and C are shown in Appendix A in Figure A1. The distinction of low and high tetragonality lift-out regions in Table 1 is based on the local tetragonality value at the indicated lift-out positions in Figure 2 and Figure A1 and is restricted to low and high due to uncertainties in the exact position.

### 3.3. APT

The APT results show a very similar average bulk carbon content for all 15 K/s tips with values between 3.2 and 3.5 at% C, similar to the nominal bulk content of 3.3 at% C, see Table 1. The 0.5 K/s reference tips have a lower average bulk carbon content with values between 2.5 and 2.9 at% C. The carbon content error determined in IVAS based on counting statistics [42] is between 0.002 and 0.009 at% C. Other alloying elements do not show any significant difference in composition or agglomeration in any of the tips.

**Figure 3:**
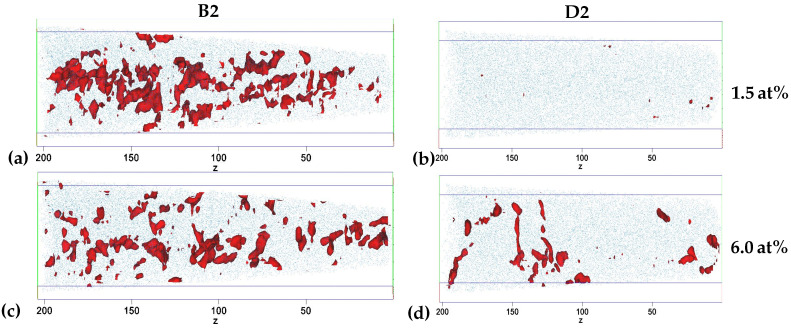
Carbon iso-concentration surfaces of tip (**a**,**c**) B2 and (**b**,**d**) D2 visualizing volumes with carbon content (**a**,**b**) below 1.5 at% and (**c**,**d**) above 6.0 at%. Fe atoms are added in blue to show the tip volume and length scale in nm.

**Figure A2:**
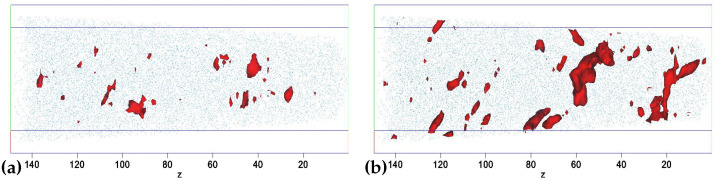
Carbon iso-concentration surfaces of tip C1 for the visualisation of volumes with carbon content (**a**) below 1.5 at% and (**b**) above 6 at% with Fe atoms added in blue to show the tip volume and length scales in nm.

The carbon iso-concentration surfaces for 1.5 at% and 6.0 at% of tips B2 and D2 are shown in Figure 3 to visualize the carbon distribution and agglomeration. The volumes enclosed by the iso-concentration surfaces are interpreted as having a low carbon content, below 1.5 at% C, and high carbon content, above 6 at% C, respectively. The B2 tip has a significant fraction of volumes with a low carbon content, while only a few very small volumes with a low carbon content are observed in D2, see Figure 3a,b. The low and high carbon volumes in B2 seem to be randomly distributed and dominantly have a bulky shape. In contrast, the high carbon volumes in D2 show clear agglomerations in two areas: 0–50 nm and around 100–200 nm. Additionally, the shape of the high carbon volumes is mostly elongated; see the area around 150 nm. The tips A1/2, B1 and C2 show a similar overall behaviour to B2 with small differences, while D1 is very similar to D2. The distribution of iso-concentration surfaces for tip C1 lies between B2 and D2 with larger high carbon volumes around 0–80 nm, while fewer and smaller volumes are observed around 80–140 nm, see Appendix B Figure A2. No significant volumes are observed for iso-concentration surfaces at 10.0 at% C in any of the samples.

**Figure 4:**
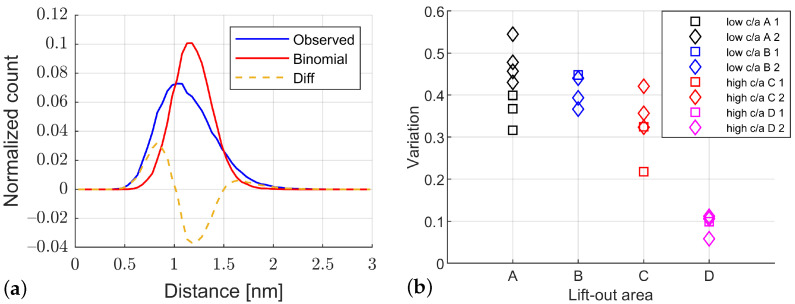
(**a**) `Observed’ and binomial normalized fifth order NN distribution with their difference in ROI of B2 with a variation value of 0.44. (**b**) Variation of fifth order NN distribution for ROI of APT tips from lift-out regions A–D with varying low and high tetragonality c/a values.

The ‘observed’ and binomial distribution of the distance to the fifth NN of all carbon ions, *C*, $C_{2}$, $C_{3}$ and $C_{4}$, along with the difference between the curves for an ROI of B2, is shown in Figure 4a. The ‘observed’ and binomial distribution for the ROI’s average bulk carbon content have a strong overlap, but a clear deviation and shift between both curves can be seen. The ‘observed’ curve is shifted to lower distances with a maximum of around 1.0 nm and has an asymmetric peak shape with a tail towards larger distances. The maximum of the binomial distribution is around 1.1 nm.

The variation *V* calculated from the fifth NN distribution with Equation (1) is shown in Figure 4b for the ROI of all regions, A–D. The results are mainly split into two groups, A–C with higher variations between 0.30 and 0.55 and D with lower variations between 0.05 and 0.11. The low variations for D show that their ’observed’ distribution has a near-perfect overlap with the binomial. The exception of the two groups is C1 ROI 2, which corresponds to the 60–120 nm area of the tip, see Figure A2, with a value of around 0.21. The ROI from the same lift-out regions have a maximal scatter of 0.11 in their variation. The same trends are observed for the other orders of the NN distribution with varying absolute values.

## 4. Discussion

The heterogeneous martensite tetragonality distribution with regions of both low and high tetragonality for the investigated 0.74 wt% C high carbon steel can be clearly seen in the EBSD results in Figure 2 and Figure A1. The asymmetry of the martensite $a_{200}$ peak in Figure 1 is an additional indication of the heterogeneous distribution of tetragonality. The presence of high tetragonality in high-carbon martensitic steel after 2 years of RT ageing is in accordance with previous observations [6].

The average bulk carbon content of the APT tips, see Table 1, shows no difference between the low and high tetragonality lift-out regions B–D, indicating that no significant autopartitioning was observed for medium carbon steels [43,44] at the cooling rates used for the 0.74 wt% C carbon steel [14] and subsequent RT ageing. Therefore, the decrease in average tetragonality during the cooling of high carbon steels [14,15] cannot be related to differences in the average carbon content on the size scale of an APT tip.

The average bulk carbon content of the 0.5 K/s reference tips, A, is significantly lower than for the 15 K/s, but this is most likely connected to the change from voltage mode to laser pulse. The fraction of molecular ions increased from 0.33–0.37 to 0.60–0.62 for the laser pulse mode, which increases the error introduced by the simplified assignment of the 24 Da peak to $C_{2}^{+}$ instead of a superposition with $C_{4}^{2+}$.

The carbon iso-concentration surfaces in Figure 3 show a clear change in carbon distribution between regions with different local martensite tetragonality, B2 and D2. The lower tetragonality region, B2, has a stronger heterogeneous distribution of carbon with larger volumes with carbon content below 1.5 at% and above 6 at% C. In contrast to this, the high tetragonality region, D2, has almost no low carbon volumes and significantly fewer high carbon volumes, indicating a more homogeneous carbon distribution. This observation is supported by the fifth NN distribution results in Figure 4 that show a clear deviation from random binomial distribution for the low tetragonality regions, A and B, while an almost perfect random distribution for the high tetragonality region, D. The results for C indicate that the lift-out is from a low tetragonality region, which will be discussed in more detail below. The counting of the molecular ions as only a single NN instead of the number of atoms in the ion is expected to influence the results but is due to the absence of spatial information for molecular ions [17,34].

The presence of carbides is indicated by the additional XRD peaks in Figure 1, but the estimated carbon content from iso-concentration surfaces is clearly below the typically expected transition η or ϵ-${\mathrm{Fe}}_{2}C$ carbides [43,45,46,47]. This is supported by the results of the fifth NN distribution in Figure 4a based on the maximum separation method due to the absence of clear indications of precipitates [35,48,49,50]. The elongated shape of the high carbon areas for D2 in Figure 3d indicates agglomeration at dislocations in high tetragonality areas as reported for as-quenched high-carbon martensitic steel [9]. Therefore, the carbide precipitation process is likely still in the stage [51] of carbon redistribution and agglomeration at lattice defects [52,53] in the high tetragonality regions, while the results for the low tetragonality regions are not conclusive, and the presence of carbides is possible. Further electron microscopy studies would be necessary for clarification, which is outside the scope of this work.

The presented results for regions A, B and D indicate that the used approach allows the correlation of local tetragonality from EBSD with the carbon distribution from APT, but the results for region C need further discussion. The tips from region C are taken from a high tetragonality region according to the EBSD measurement, see Figure A1b, but C2 has a very similar carbon distribution as the APT tips from the low tetragonality region, A and B, while C1 shows a mixed character. The area from 0 to 60 nm of C1 is very similar to the low tetragonality results of A and B, while the area from 60 to 120 nm shows a behaviour between the iso-concentration surfaces as well as NN distribution, see Figure 4b and Figure A2. There are multiple possible explanations for the bad correlation between the tetragonality and APT results for region C. First of all, EBSD is a surface-sensitive technique with expected depth information around 60 nm in Fe at 20 kV based on results for Cu [54] that has a similar atomic number, while the final analysed volume in APT is not directly related to the surface due to the lift-out procedure [24,25]. The exact depth depends on the FIB milling process, and the depth of the possible implantation of Ga needs to be taken into account [55]. Additionally, the used EBSD step size of 75 nm is similar to the diameter of the analysed APT tips. Therefore, the local tetragonality from EBSD can only be used as an indication of the martensite tetragonality in the tip, while the exact distribution of local tetragonality in the tip is not accessible with this approach.

Finally, the local correlation of the tetragonality and carbon distribution opens a few interesting questions about the origin of tetragonality in martensite. The tetragonality is generally attributed to the average bulk carbon content, and the correlation of both has been studied in great detail [1,2,7,8,9], but the possible effect of residual strains from the martensitic transformation has been discussed as well [56]. The previous experimental results have been limited to a comparison of one global parameter, typically the tetragonality determined by XRD, with the global [5] and/or local [8,9] distribution of the other parameter, which is typically the carbon content. For example, Tanaka et al. [8] compared the local tetragonality with the global carbon content. Thus, this is the first time, to the authors’ knowledge, carbon content and tetragonality have been correlated locally, even though it is only an approximation. The results show a clear mismatch between the average bulk carbon content and the expected tetragonality. The low tetragonality lift-outs have a similar average bulk carbon content as the nominal content of 3.3 at% C, see Table 1, while the expected carbon content for a tetragonality of around 1.01 as for lift-out region A, see Figure 2a, would be around 1.0 at% [1] or 1.5 at% C [7] depending on the used equation. This difference can partially be explained by the significant fraction of volumes with low carbon content in Figure 3a, but it is not clear how the variation of high and low carbon content on the scale of tens of nm impacts the local tetragonality determined by EBSD since it would be an average over the complete width of the tip due to the used step size and interaction volume. The mismatch of local tetragonality and average bulk carbon content of an APT tip indicates that local carbon variations and internal strains could be an important contribution to martensite tetragonality.

## 5. Conclusions

The applied novel approach allows the correlation of local martensite tetragonality measured by electron backscatter diffraction and carbon distribution by atom probe tomography (APT) in a heterogeneous martensite microstructure of high carbon steel on an nm scale.

It is shown that regions with a low tetragonality have a stronger heterogeneous carbon distribution compared to regions with a high tetragonality. The iso-concentration surfaces show clear local agglomeration of carbon and the presence of areas with low carbon content for lower tetragonality. Regions with high tetragonality only have a few elongated agglomerations with high carbon content, possibly along dislocations. The statistical nearest neighbour analysis confirms the stronger heterogeneous carbon distribution for regions with a low tetragonality, while regions with a high tetragonality show a nearly random distribution.

No quantitative differences in the average bulk carbon content of the APT tips are observed between low and high tetragonality regions.

## Figures and Tables

**Table 1:** Overview of final cooling rate, ageing time, mean expected diffusion distance $x_{i}$, local martensite tetragonality c/a, average bulk carbon content and number of ROI for lift-out regions A–D and the two respective APT tips per region.

Lift-out region	A		B		C		D	
Final cooling rate	0.5 K/s		15 K/s					
RT ageing time (days)	570		727		823		823	
$x_{l}$ (nm) for $D_{l}$	50		57		60		60	
$x_{u}$ (nm) for $D_{u}$	446		503		535		535	
c/a EBSD	low		low		high		high	
APT tip name	A1	A2	B1	B2	C1	C2	D1	D2
Avg. bulk comp. C (at %)	2.9	2.5	3.5	3.2	3.3	3.2	3.3	3.3
Number of ROI	3	4	1	3	2	3	1	3

## Data Availability

The raw data cannot be shared at this time as the data are also part of an ongoing study.
